# Forty Years Trends in Timing of Pubertal Growth Spurt in 157,000 Danish School Children

**DOI:** 10.1371/journal.pone.0002728

**Published:** 2008-07-16

**Authors:** Lise Aksglaede, Lina W. Olsen, Thorkild I. A. Sørensen, Anders Juul

**Affiliations:** 1 University Department of Growth and Reproduction, Rigshospitalet, Copenhagen, Denmark; 2 Institute of Preventive Medicine, Copenhagen University Hospital, Centre for Health and Society, Copenhagen, Denmark; Center for Research for Mothers & Children (CRMC), United States of America

## Abstract

**Background:**

Entering puberty is an important milestone in reproductive life and secular changes in the timing of puberty may be an important indicator of the general reproductive health in a population. Too early puberty is associated with several psychosocial and health problems. The aim of our study was to determine if the age at onset of pubertal growth spurt (OGS) and at peak height velocity (PHV) during puberty show secular trends during four decades in a large cohort of school children.

**Methods and Findings:**

Annual measurements of height were available in all children born from 1930 to 1969 who attended primary school in the Copenhagen Municipality. 135,223 girls and 21,612 boys fulfilled the criteria for determining age at OGS and age at PHV. These physiological events were used as markers of pubertal development in our computerized method in order to evaluate any secular trends in pubertal maturation during the study period (year of birth 1930 to 1969). In this period, age at OGS declined statistically significantly by 0.2 and 0.4 years in girls and boys, respectively, whereas age at PHV declined statistically significantly by 0.5 and 0.3 years in girls and boys, respectively. The decline was non-linear with a levelling off in the children born between 1940 and 1955. The duration of puberty, as defined by the difference between age at OGS and age at PHV, increased slightly in boys, whereas it decreased in girls.

**Conclusion:**

Our finding of declining age at OGS and at PHV indicates a secular trend towards earlier sexual maturation of Danish children born between 1930 and 1969. Only minor changes were observed in duration of puberty assessed by the difference in ages at OGS and PHV.

## Introduction

Entering puberty is an important milestone in reproductive life, and changes in the timing of puberty have been an area of great research interest for decades because of its associated health and psychosocial problems [1]. A declining age at menarche has been observed in industrialized European countries, and in the US, over the last 100 years until the middle of the 20th century where this trend seemed to cease, probably due to the increasing stability in socio-economic conditions, nutritional status and hygiene (for review see [2]). However, recent data on earlier maturation among US girls [3]–[5] compared with previous studies [6]–[8] have created new and increasing concerns and a demand for worldwide monitoring of puberty. In the American studies, age at breast development in girls had declined significantly, but a similar decline in age at menarche was not detected [3]–[5]. This suggests that the duration of the pubertal transition has increased in that same period. Timing of puberty is under tight genetic control as illustrated by a high heritability of age at menarche [9]. However, genetic variation cannot explain this rapid change in timing of puberty over just a few decades, suggesting that environmental factors must be involved.

The most important physiological hallmarks of puberty are development of secondary sexual characteristics and pubertal growth spurt, phenomena that are closely interrelated. There is much variation in the timing of the onset of puberty among both girls and boys, but, within each sex, the overall sequence of pubertal events is well preserved [10]. Thus, the pubertal growth spurt is an early pubertal event in girls, which occurs soon after the initiation of breast development in the majority, whereas, in boys, the growth spurt occurs as a later pubertal phenomenon about one year after pubertal onset, defined by an increase in testicular size [10], [11]. Therefore, growth may serve as a tool in the assessment of secular changes in pubertal timing, but must be considered separately for girls and boys, respectively.

Secular trend analyses, based on existing data on sexual maturation, are often limited by data comparability among studies in different populations, different periods of time, and the use of different methods, such as palpation vs. inspection of breast development etc [2], [12]. As a result, conclusions from data comparisons may not be consistent. Furthermore, most studies on pubertal timing report only on changes in age at menarche in girls, whereas limited data exist on pubertal timing in boys. Moreover, as a retrospective information age at menarche is, by nature subject to possible recall bias. It is currently debated, and remains an open question, whether there is a decline in pubertal timing of quantitative significance [13].

We therefore analysed the timing of puberty in a large cohort of Danish school children over four decades as assessed by age at onset of growth spurt (OGS) and age at peak height velocity (PHV) during puberty to determine if a secular trend was evident. In this unique material of more than 150,000 children, we utilized standardized indicators of pubertal onset and progression. Furthermore, we aimed at analysing the duration of puberty as assessed by the time between OGS and PHV and to see if there was any difference in a potential secular trend between boys and girls.

## Materials and Methods

Our study was based on a cohort of 149,992 girls and 151,604 boys born between 1930 and 1969 who had undergone mandatory annual health examinations in public or private primary schools in the Copenhagen Municipality.

### Measurements

The school health records included measurements of height (measured to the nearest 1 cm), date of birth, and date of measurement (year and month), from which data have been computerized [14], [15]. The children were measured during all the years of school attendance, and the measurements were mandatory; all children were measured each year. Throughout the entire period, measurements were performed on the children wearing no shoes.

### Subjects

Subjects eligible for this study were children born 1930 through 1969, who had a minimum of five height measurements recorded on the school health record. We further limited the analysis to girls who had at least one height measurement recorded in their thirteenth year or later, and to boys who had a height measurement recorded in their fifteenth year or later.

From July 1984, children were regularly examined only when they entered and left school. Thus, additional ad hoc measurements after 1983 were excluded to prevent any potential bias that may have arisen through non-systematic exams. Some children were measured more frequently than others. To ensure homogeneity among the measurements, we replaced measures with the average if there were two or three measurements recorded with a frequency of four months or less.

### Criteria and definitions

Based on growth patterns obtained in a longitudinal study of healthy boys and girls [16], [17], we designed the following criteria. Height velocity was defined as the difference in heights at two adjacent measurements, divided by the difference in time, and assigned this value at an age in the middle of this period. Age at PHV was defined as the age at maximal height velocity after the age of 8.00 years for girls, and after the age of 10.00 years for boys. Age at OGS was defined as the ‘latest’ minimum before age at PHV. A minimum was defined as a rate of change of the height velocity (the 2nd derivative) less than 0.5 cm per year. If the difference between PHV and height velocity at OGS was less than 2 cm/year for girls or less then 2.5 cm/year for boys, we categorized age at PHV and OGS as missing, since the lack of intra-individual variation in the growth velocity over time in this age seems unlikely. Furthermore, if the age at PHV coincided with the last recorded height velocity, the age at PHV was defined only if the velocity exceeded 8 cm/year for girls and 10 cm/year for boys at this point, respectively. Likewise, if age at OGS coincided with the first recorded height velocity, the age at OGS was defined only if velocity was lower than 6 cm/year.

Among the missing values of age at OGS and PHV, we defined these ages as having not yet occurred if a decline in height velocity was not observed, i.e. if height velocity exceeded 4 cm/year at the last recorded measurement. Otherwise, we defined the missing values as if OGS and PHV had occurred within the observed age range, but that the yearly measurement was too sparse to determine age at OGS and at PHV.

Application of these criteria and definitions reduced the population of girls for analysis from 149,992 girls to 135,223 and boys from 151,604 to 21,612.

### Statistical analysis

Time trends in age at OGS and at PHV were analyzed using regression analysis with age at OGS and age at PHV, respectively, as dependent variables and time categorized into 5-year categories as explanatory variable. The two types of censoring, left (if the child had not reached puberty before leaving school) and interval (if the child had stopped growing when leaving school, but the yearly measurement left us unable to determine the age at OGS or PHV) were taken into account using SAS' proc lifereg procedure. This fits regression models with normally distributed error terms to failure time data, which can be right, left or interval censored. These models are then basis for tests of the null hypotheses of no secular trends and for estimation of the confidence intervals. We calculated the difference between the estimate for age at PHV and age at OGS. We obtained approximate confidence limits and p-values for the difference from the estimated standard error on age at OGS and age at PHV. The data structure with censoring implied that the correlation between individual measures of age at PHV and age at OGS could not be taken into account, and, therefore, these confidence limits and p-values will tend to be conservative, i.e. with wider confidence intervals and higher p-values than otherwise would have been obtained.

### Validation of computational method

The method by which we determined age at OGS and age at PHV was validated on 20 boys and 20 girls from our endocrine outpatient clinic who were followed closely with regular measurements of height during childhood and adolescence, and who fulfilled the inclusion criteria. Ages at OGS and at PHV were defined visually, and for each child separately, by a trained paediatric endocrinologist on graphs of the complete sets of measurements as well as computerized on a subset of randomly selected annual measurements.

These annual measurements were randomly selected in order to create a data set comparable to the one based on the annual measurements from the school health records. The person who performed the visual assessment was blinded from the computer-based assessment. In order to examine if the computation of age at OGS and PHV leads to any systematic upward or downward bias, the difference between the two determinations was compared by a paired t-test. We found no statistical difference between the computerized ages at OGS and PHV and the manually defined ages (p>0.05 in all cases). In girls, the mean difference between computerized and manually defined ages at OGS and PHV was −0.13 years (CI −0.61 to 0.35) and 0.28 years (CI −0.12 to 0.68), respectively. Likewise, in boys, the difference between computerized and manually defined OGS and PHV was 0.13 years (CI −0.11 to 0.38) and −0.17 years (CI −0.43 to 0.088), respectively.

**Figure 1:**
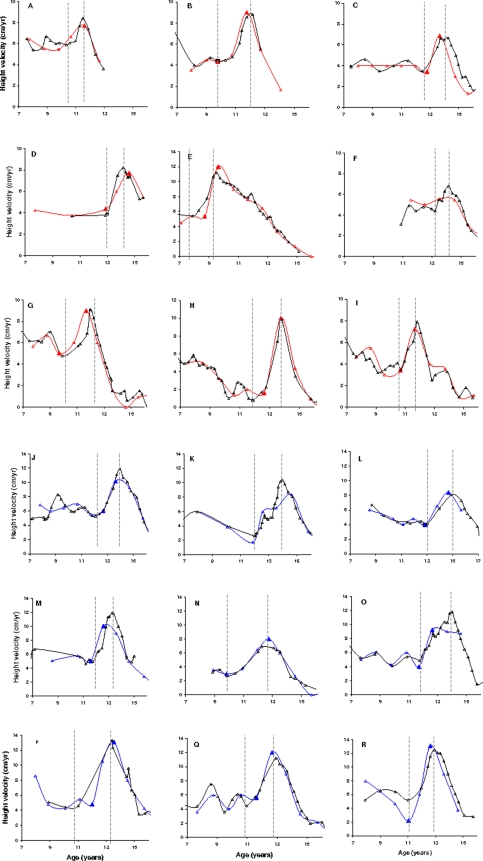
Height velocity curves in 9 girls and 9 boys from the validation of the method. Height velocity curves in a subset of 9 girls (A–I) and 9 boys (J–R) from the validation of the method. The complete set of measurements and the randomly selected annual measurements are illustrated with indications of age at onset and PHV judged by both visual and computerized methods independent of each other. Note Figure 1F and 1K, where ages at onset and PHV were not determined by computation. These children were recorded as having reached onset of pubertal growth and PHV, but the yearly measurement was too sparse to determine the pubertal growth. By looking at the complete set of measurements there was no doubt that all these children had reached onset and PHV and the interval censoring of these children was thus fully acceptable.

The defined age at OGS and age at PHV in these 40 subjects with multiple measurements of height, and the computerized values on annual measurements in the same subjects, showed a correlation coefficient for both age at OGS and at PHV of 0.81 and 0.79, respectively, in girls, and 0.92 and 0.91, respectively, in boys (all p<0.0001). A randomly selected subset of growth velocity charts on 9 boys and 9 girls out of these 40 subjects are shown in Figure 1. The complete set of height measurements and the randomly selected annual measurements are illustrated with indications of age at OGS and PHV judged by both manual and computerized methods. In three girls and three boys out of the 40 subjects, ages at OGS and PHV could not be determined by computation and these children were recorded as having reached OGS and PHV, but yearly measurements were too sparse to determine the exact age in these subjects. These children were thus interval censored. By looking at the complete set of measurements in an example of this phenomenon (Figures 1F and 1K) there was no doubt that these children had reached OGS and PHV and the censoring was thus fully acceptable.

### Ethics

Since the study is entirely based on register data, there is no request for an ethical permission, but only an approval by the Danish Data Protection Agency, which was obtained.

## Results

156,835 children (boys; n = 21,612 and girls; n = 135,223) fulfilled the inclusion criteria for this study and in a total of 101,118 children (boys; n = 14,689 and girls; n = 86,429) age at OGS and at PHV was determined (Table 1). The number of boys fulfilling the inclusion criteria (n = 36) in the first five years (1930–1935) was very limited since school attendance was only obligatory for 7 years at that time, and most of the boys thus left school before entering puberty. Boys born before 1935 were therefore excluded from the analysis (Table 1). Boys were measured 9.1 times, whereas the mean number of measurements in girls was 8.4.

**Table 1:** Subjects included in the study.

Year of birth	Study cohort		No fulfilling inclusion criterion		No with termination of OGS and PHV		Total no of individual measurements	
	Girls	Boys	Girls	Boys	Girls	Boys	Girls	Boys
**1930–34**	18.029	18.364	13.667	36	7.873	*26*	109.666	347
**1935–39**	23.323	23.683	19.657	75	12.373	49	162.058	697
**1940–44**	27.055	27.302	24.972	306	15.333	197	196.412	3.077
**1945–49**	26.150	26.301	24.934	408	16.406	283	215.495	4.039
**1950–54**	18.483	18.432	17.739	2.758	11.661	1.997	157.45	27.131
**1955–59**	14.524	14.815	13.777	9.879	9.342	6.771	122.483	91.433
**1960–64**	11.824	11.886	11.047	3.555	7.278	2.38	94.632	30.978
**1965–70**	10.604	10.821	9.43	4.595	6.163	2.986	80.056	39.889
**Total**	**149.992**	**151.604**	**135.223**	**21.612**	**86.429**	**14.689**	**1,138,252**	**197.591**

In both girls and boys, we found a significant decline in the age at OGS and age at PHV over the four decades studied, suggesting a secular trend towards earlier sexual maturation of Danish children born between 1930 and 1969. On average, girls born in 1930–35 reached the OGS and PHV at 10.4 and 12.5 years, respectively, whereas girls born in 1965–70 reached OGS and PHV at 10.1 and 12.0 years, respectively. Likewise, on average, boys born in 1935–40 reached the OGS and PHV at 12.2 and 14.5 years, respectively, whereas boys born in 1965–69 reached OGS and PHV at 11.8 and 14.2 years, respectively.

**Figure 2:**
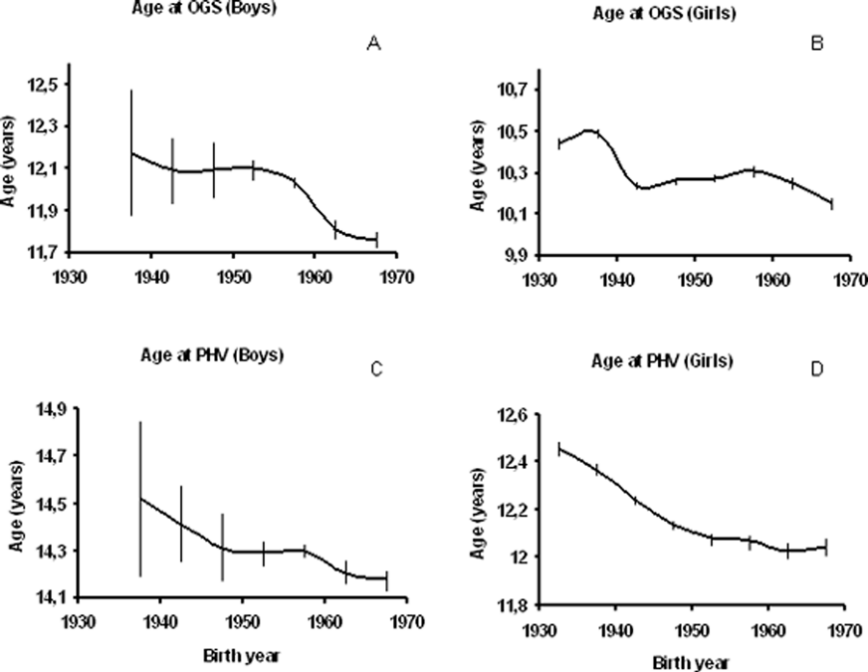
Trends in age at OGS and PHV. Age at onset of pubertal growth spurt (Age at OGS) in boys (A) and girls (B), and age at peak height velocity (Age at PHV) in boys (C) and girls (D) according to year of birth. Curves are smoothed by 5-year intervals (e.g. 1930–1934, 1935–1939, 1940–1944 etc.) and presented with confidence intervals.

The secular trend of the estimates applied in five-year intervals is shown in Figure 2. The decline in age at OGS in both boys and girls and in age at PHV in girls was non-linear (all p<0.0001), whereas the age at PHV in boys did not differ significantly from a linear decline. We found a tendency towards a levelling off in the declining age at pubertal maturation in the children born between 1940 and 1955.

The duration of puberty, as defined by the difference between age at OGS and age at PHV, increased from 2.3 to 2.4 years in boys born between 1935–1969. In girls, in contrast, duration declined from 2.0 to 1.9 years during the study period (Table 2).

**Table 2:** Duration of puberty.

	Year of birth	Age at OGS (yrs)	Age at PHV (yrs)	Duration of puberty* (yrs)	Confidence interval	p-value**
Girls	1930–34	10.4	12.5	2.0	(1.98–2.05)	0.00003
	1935–39	10.5	12.4	1.9	(1.85–1.91)	0.76
	1940–44	10.2	12.2	2.0	(1.98–2.03)	0.00001
	1945–49	10.3	12.1	1.9	(1.85–1.90)	0.61
	1950–54	10.3	12.1	1.8	(1.77–1.83)	0.002
	1955–59	10.3	12.1	1.8	(1.72–1.79)	0.00001
	1960–64	10.3	12.0	1.8	(1.73–1.82)	0.0004
	1965–70	10.2	12.0	1.9	(1.84–1.93)	1.0
Boys	1935–39	12.2	14.5	2.3	(1.90–2.79)	0.76
	1940–44	12.1	14.4	2.3	(2.10–2.54)	0.42
	1945–49	12.1	14.3	2.2	(2.03–2.41)	0.057
	1950–54	12.1	14.3	2.2	(2.12–2.27)	0.000001
	1955–59	12.0	14.3	2.3	(2.22–2.30)	0.00002
	1960–64	11.8	14.2	2.4	(2.34–2.47)	0.72
	1965–70	11.8	14.2	2.4	(2.36–2.47)	1.0

* Difference between age at OGS and at PHV. **For comparison with the last value in girls and boys, respectively.

**Figure 3:**
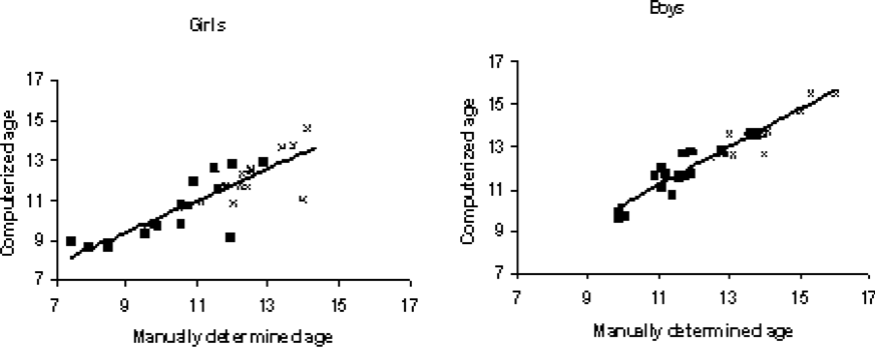
Validation of the method. Correlation between manually determined and compute-based estimates of the age at OGS (as indicated by ▪) and at PHV (as indicated by x) in 20 girls (left panel) and 20 boys (right panel) with multiple height-measurements.

Confidence intervals for the estimates of ages at OGS and PHV are presented in figure 3.

## Discussion

We hereby describe a secular trend in pubertal maturation in our longitudinal study on 135,223 girls and 21,612 boys born 1930 through 1969, as evidenced by a significant decline in the age at OGS and at PHV. Our computerized method for describing two well-defined events of the adolescent growth (OGS as an early pubertal event, and PHV as a late pubertal event) gives us the opportunity to look back in time and determine any possible secular trends in these distinct markers of age at sexual maturation in our large population-based cohort. Girls born in 1969 reached OGS and PHV 0.3 and 0.5 years earlier than girls born in 1930, respectively, whereas boys born in 1969 reached the same milestones 0.4 and 0.3 years earlier, respectively, compared to boys born in 1935.

Most studies on pubertal timing report only on changes in girls, whereas data in boys are sparse. This is most probably due to the relatively easy access to information on age at menarche, whereas, in boys, no such characteristic event in puberty exists, and clinical examination of boys is usually required for such assessment. In Denmark, the mean menarcheal age has declined from approximately 17 years in the 19^th^ century to approximately 13–13.5 years [18]. In studies of birth cohorts from the same period, as described in the present study, age at menarche declined from 13.8 years to 13.0 years [19], [20], which is supported by our finding of a declining age at puberty in girls born between 1930 and 1969. Interestingly, the tendency towards earlier sexual maturation in Denmark seemed to cease in studies performed after the present study [21]–[23].

Assessment of age at OGS and at PHV based on growth charts has previously been carried out in a number of studies. Some of these were based on individual visual estimates, whereas other groups developed methods for determining these ages by computation. Persson et al [24] adapted a manual method (“the three-ruler method”) as previously described by Karlberg et al [25] in order to determine age at OGS [24]. Three connected rulers were fitted to the height measurements to estimate the age at which the curve started to deviate by more than 12 degrees, representing a growth velocity of >6 cm/year and marking the onset of puberty [24].

For population-based studies in a larger scale, however, it is evident that a computerized estimation is more convenient and more reliable. Already in 1825 the equation of Benjamin Gompertz was developed [26], which was much later described as applicable to the growth of biological organisms (for review see [27]). The Preece-Baines model is an automated mathematical method for describing normal growth from a given age before adolescence until adulthood [28]. This method is based on non-linear least square techniques and has been used to describe the adolescent growth in several studies [29]–[34].

In a recent study on Indian children and adolescents, a decline in the age at OGS and age at PHV in girls over four decades (1950–89) was found, whereas the same parameters were constant in boys [35]. A structural analysis (the Bock-Thissen-di Toit model) was used for deriving the average curves in this study.

Taranger et al [36] calculated height increments over periods of 12 months, and defined age at PHV as the age at the midpoint in the interval with the greatest increment [36]. Their study was based on standing height measurements every third month giving the opportunity to evaluate the value of 6 and 12 months measurements, respectively, for the determination of PHV compared to measurements every 3 months. Taranger et al found that the 6 and 12 months estimates were less precise and carried a risk of underestimating the age at PHV as compared to the 3-month estimates [36]. This contrasts with our finding of a very high accordance between estimated age at PHV based on 12-month measurements and 3–4-monthly measurements (Figures 1 and 3).

Estimated peak mean annual height increment, as defined by the mean increase in height in each successive year, was compared between different cross-sectional studies from Greece in the period between 1928 and 1995 [37]. The author found a tendency toward earlier sexual maturation in girls but no such secular trend in boys. However, this study was based on cross-sectional data and, even though at least 100 children were included in each age group and each sex, it is very difficult to conclude on height increment since all measurements were from different children. Our method for determining age at the important pubertal milestones related to the pubertal growth spurt is fast and valid. Our method is applicable to large population-based studies where individual visual determination of these puberty markers is not feasible.

In the early 1990ies, much earlier breast development in American girls was reported in two large epidemiological studies (PROS and NHANES III) [3]–[5] compared with previous US studies from 1948–74 [6]–[8]. These studies did, however, not detect a similar decline in age at menarche, and it seemed that the age at onset of puberty had changed but not the progression through later puberty stages [3], [38], [39]. It has been widely debated if these contradictory observations could be a result of an underestimated age at breast development in obese girls or if a prolongation of the entire pubertal transition was present [13]. Discrimination of breast (glandular) tissue from fat is very difficult, and especially in overweight or obese girls with excessive subcutaneous fat, palpation is necessary. Part of the PROS study was based on breast palpation (39% of subjects) [40], and reanalysis of the data including data on palpation confirmed the previous finding [40]. In our study, we found a tendency towards longer duration of puberty as defined by increasing difference between the age at OGS and age at PHV (an early and a late pubertal event) in boys only. By contrast, we found a reduced duration of puberty in girls during the four decades studied. However, it has to be emphasised that the difference was only 0,8 and −1.5 months, in girls and boys, respectively, during the study period of 40 years.

The ability to adequately compare two or more studies performed at different points in time is affected by the differences in the study designs and study populations. For example, newer studies may have used more reliable methods (e.g. breast palpation), which differ from methods used in an older study. Thus, secular trend analysis may be limited, and, as a result, conclusions from data comparisons may not be consistent.

Our finding of a decline in age at maturation in children born between 1930 (1935 in boys) and 1969 may indicate that some environmental factors may affect the whole population. It is plausible to hypothesise that the Second World War influenced the prenatal or early life environmental factors of these birth cohorts. It has previously been shown that World War II postponed age at menarche in several European countries [41], [42]; a tendency causally linked with circumstances of general famine and food rationing. However, in Denmark the general population was not affected by food restrictions as was the case in other European countries, and as previously reported no effect on growth in Danish children was found during these years [43].

Endocrine disrupting chemicals (EDC) from the environment has been shown to influence puberty timing in animal studies as well as in wildlife observations [44]. However, little is known about the possible role of EDCs for the timing and progression of human puberty. Because of our modern way of living humans are exposed to a wide variety of suspected EDCs but the exposure levels may be low, the potency of the compounds weak, and clear effects on endocrine function from such exposures are therefore difficult to demonstrate. However, prepubertal children are highly responsive to sex steroid actions. Because the endogenous levels of sex steroids in prepubertal children are very low, even a small variation may account for a major change in the total activity of the involved hormone, which is reflected in phenotypic effects in the child [45].

Whether or not the initial secular decline in age of the pubertal growth and also of the corresponding sexual maturation, indicated by age at menarche, has been associated with changes in psychosocial functioning and health status in general remains to be investigated.

The main strength of our study is the availability of computerized, annually collected anthropometric measures in a large population-based sample of children from the same geographic area over 4 decades. The data has been handled equally at all time points and the risk of erroneous conclusions due to different study designs is minimal. The present study included all children who attended primary, public or private, school in the Copenhagen Municipality during a 40-year period. Theoretically, it can be speculated that young subjects with relatively early onset, or older children with no signs of puberty, would decline to participate in a survey on puberty. However, these health examinations were mandatory and performed in all schools during the study period, and we consider that the potential risk for such selection bias is small. After 1983, regular health examinations were only performed at entering and leaving school. In order to avoid bias on children with some physical condition requiring closer follow up, e.g. obesity or pubertal disorders we excluded all measurements after 1983. The number of children included in our study was reduced from 149,992 girls to 135,223, and 151,604 boys to 21,612, respectively, after application of our mathematical criteria for determining age at OGS and at PHV. Thus, the withdrawal of girls was only minor, whereas in boys the reduction was more pronounced. The reason for this difference between the sexes may be that school attendance was only mandatory for 7 years until 1972 in Denmark. One of our criteria was measurement in each girl after the age of 13 years and after the age of 15 years in boys, and many boys, but not girls, thus left school before these ages. We cannot exclude that the high proportion of growth charts from boys in which OGS and PHV could not be validly determined by our method, especially in the early decades studied, could pose a theoretical selection bias. However, the selection of children was systematic and not based on social class e.g., which might bias the results of our study. Data on the auxological instruments used in each individual school was not reported, but we consider the uncertainty related to the potential inter-observer variation as limited due to the high number of children participating in this study.

Our study is limited by lack of detailed information on health status or nutrition in each child. However, the nutritional supplies in Denmark and Copenhagen were adequate throughout the time period of the study, and the occurrence of diseases in this population of children of school age was expectedly very low and unlikely to affect the statistics of this study. Even though the Danish society in this period was based on equity principles for its citizens and a strong tendency toward social equality, there was of course social gradients that have changed over time and which might have influenced the timing of pubertal growth.

In conclusion, we found a significant downwards secular trend in the timing of pubertal maturation in Danish children born between 1930 (1935 in boys) and 1969 as evidenced by a decline in the age at which the onset of pubertal growth spurt and age at peak height velocity occurred. The difference between age at OGS and age at PHV increased in boys, but not in girls, during the study period indicating that the duration of puberty was longer in the latest cohort of boys compared to the cohort born in 1935. The described change in timing of puberty is noteworthy because of the possible associated health and psychosocial problems.
